# Draft genome assemblies of the ponerine ant *Odontoponera transversa* and the carpenter ant *Camponotus friedae* (Hymenoptera: Formicidae)

**DOI:** 10.1186/s12863-024-01253-7

**Published:** 2024-07-15

**Authors:** Jinlin Liu, Zijun Xiong, Youliang Pan, Jie Zhao, Wei Dai, Qunfei Guo, Weiwei Liu, Qiye Li

**Affiliations:** 1 School of Biology and Biological Engineering, South China University of Technology, Guangzhou, China; 2 BGI Research, Wuhan, 430074 China; 3 State Key Laboratory of Genetic Resources and Evolution, Kunming Institute of Zoology, Chinese Academy of Sciences, Kunming, China; 4 Yunnan Key Laboratory of Bioaffiliationersity Information, Kunming, China; 5 College of Life Sciences, University of Chinese Academy of Sciences, Beijing, China

**Keywords:** Long-read sequencing, *Odontoponera*, *Camponotus*, Genome assembly, Annotation

## Abstract

**Objectives:**

Ants are ecologically dominant insects in most terrestrial ecosystems, with more than 14,000 extant species in about 340 genera recorded to date. However, genomic resources are still scarce for most species, especially for species endemic in East or Southeast Asia, limiting the study of phylogeny, speciation and adaptation of this evolutionarily successful animal lineage. Here, we assemble and annotate the genomes of *Odontoponera transversa* and *Camponotus friedae*, two ant species with a natural distribution in China, to facilitate future study of ant evolution.

**Data description:**

We obtained a total of 16 Gb and 51 Gb PacBio HiFi data for *O. transversa* and *C. friedae*, respectively, which were assembled into the draft genomes of 339 Mb for *O. transversa* and 233 Mb for *C. friedae*. Genome assessments by multiple metrics showed good completeness and high accuracy of the two assemblies. Gene annotations assisted by RNA-seq data yielded a comparable number of protein-coding genes in the two genomes (10,892 for *O. transversa* and 11,296 for *C. friedae*), while repeat annotations revealed a remarkable difference of repeat content between these two ant species (149.4 Mb for *O. transversa* versus 49.7 Mb for *C. friedae*). Besides, complete mitochondrial genomes for the two species were assembled and annotated.

## Objective

Ants are group-living insects that exhibit complex social behaviors, obligate reproductive division of labor via queen–worker caste differentiation, and extended queen longevity [1]. The common ancestor of all modern ants was estimated to appear in the late Jurassic, and the stem-group ants experienced species radiation during the Early Cretaceous, giving rise to the species-rich Formicidae with > 14,000 extant species in about 340 genera [2, 3]. Moreover, these tiny creatures have adapted to most terrestrial environments, where they are usually the most abundant insects in local ecosystems [4]. The ants therefore represent a unique system to study the genetic underpinnings of species radiation and adaption as well as the evolution of developmental plasticity and longevity. This is particularly true in the genomic era, when considering that ant genomes are generally small (200–600 Mb) [5]. Indeed, a large-scale genome sequencing initiative has been proposed for ant genomics, i.e., the Global Ant Genomics Alliance (GAGA) [4]. And thanks to the efforts of scientists around the world, the genomes of > 120 ant species have been sequenced and assembled to date. However, species selected for genome sequencing were mainly collected in Europe and America and are under-represented in some other regions, such as East and Southeast Asia. We contemplate that the uneven collection of ant genomes, in terms of geographical representation, will limit the in-depth investigation of ant phylogeny and genomic evolution.

In this study, we would like to mitigate this unevenness by conducting whole genome sequencing for two ant species with a natural distribution in China, the ponerine ant *Odontoponera transversa* and the carpenter ant *Camponotus friedae*. *O. transversa* is commonly observed in Southeast Asia and South China [6, 7]. It is a predator that feeds on small insects, especially termites [8]. It has been reported that *O. transversa* can use the termite trail pheromone to track the termites [9]. The predation behavior of *O. transversa* is believed to play an important role in preventing the disaster of some pests and maintaining ecological balance [10]. It is also noteworthy that *O. transversa* represents one of the only two extant species in the genus *Odontoponera* [7]. In contrast, *C. friedae* belongs to a species-rich genus that encompasses over 1,500 species [11]. The geographic range of *C. friedae* is mainly restricted to the eastern part of mainland China, Taiwan and Japan [6]. Colonies of *C. friedae* are typically monogynous with a single queen, and the workers are polymorphic with major and minor workers [12]. The two genome assemblies obtained in this study represents the first reference genome for the genus *Odontoponera*, and the fifth one for the species-rich genus *Camponotus*. Therefore, we anticipate that these genomic resources will provide valuable genetic resources for understanding the biology of *Odontoponera* and *Camponotus*, and also facilitate future study of ant phylogeny and evolution.

## Data description

The samples of *O. transversa* were collected from woodland near Yangmei Middle School (21°32′57.26″N, 110°36′39.71″E), Huazhou City, Guangdong Province in March 2020. The *C. friedae* samples were collected from the mountain adjacent to Shantang village (26°13′17.04″N, 119°34′12.44″E), Fuzhou City, Fujian Province, also in March 2020. After collection, the ants were immediately transferred to the lab at Kunming Institute of Zoology, Yunnan, China, where they were frozen in liquid nitrogen and subsequently stored at -80 °C. The samples were carefully packaged with dry ice and sent to Novogene Corporation (Tianjin, China) for DNA extraction and genome sequencing.

For each species, genomic DNA (gDNA) was extracted from a pool of multiple individuals with a sodium dodecyl sulfate (SDS) based method implemented by Novogene. Specifically, eight *O. transversa* workers and 10 *C. friedae* gynes were pooled respectively before DNA extraction. Then gDNA was fragmented to a target size of approximately 15 kb and subjected to the construction of PacBio HiFi SMRTbell libraries with the SMRTbell Express Template Prep Kit 2.0 according to the manufacturer’s instructions (Pacific Biosciences, CA, USA). The HiFi reads were produced using the circular consensus sequencing (CCS) mode on the PacBio Sequel II System (Table 1, Data set 1). We obtained a total of 16 Gb and 51 Gb HiFi reads for *O. transversa* and *C. friedae*, respectively (Data file 1).

**Table 1:** Overview of data files/data sets

Label	Name of data file/data set	File types (file extension)	Data repository and identifier (DOI or accession number)
Data file 1	Statistics of PacBio HiFi data	Spreadsheet (.xlsx)	Figshare, 10.6084/m9.figshare.25108073. [26]
Data file 2	Statistics of RNA-seq data	Spreadsheet (.xlsx)	Figshare, 10.6084/m9.figshare.25108073. [26]
Data file 3	Statistics of genome assembly	Spreadsheet (.xlsx)	Figshare, 10.6084/m9.figshare.25108073. [26]
Data file 4	BUSCO assessment of genome assembly	Spreadsheet (.xlsx)	Figshare, 10.6084/m9.figshare.25108073. [26]
Data file 5	Read depth of the assemblies	Spreadsheet (.xlsx)	Figshare, 10.6084/m9.figshare.25108073. [26]
Data file 6	Statistics of gene annotation	Spreadsheet (.xlsx)	Figshare, 10.6084/m9.figshare.25108073. [26]
Data file 7	Statistics of repeat annotation	Spreadsheet (.xlsx)	Figshare, 10.6084/m9.figshare.25108073. [26]
Data set 1	PacBio HiFi sequencing reads	Bam file (.bam)	NCBI, https://identifiers.org/ncbi/insdc.sra: SRP509859. [27] CNGB, https://db.cngb.org/search/project/CNP0005508. [28]
Data set 2	RNA-seq reads	Fastq file (.fastq)	NCBI, https://identifiers.org/ncbi/insdc.sra: SRP509859. [27] CNGB, https://db.cngb.org/search/project/CNP0005508. [28]
Data set 3	Genome assemblies	Fasta file (.fasta)	NCBI, https://identifiers.org/ncbi/bioproject: PRJNA1114375. [29] CNGB, https://db.cngb.org/search/project/CNP0005508. [30]
Data set 4	Protein-coding gene annotations of *O. transversa*	Gff file (.gff)	Figshare, 10.6084/m9.figshare.25108073. [26]
Data set 5	Protein-coding gene annotations of *C. friedae*	Gff file (.gff)	Figshare, 10.6084/m9.figshare.25108073. [26]
Data set 6	Functional annotations of *O. transversa*	Lis file (.lis)	Figshare, 10.6084/m9.figshare.25108073. [26]
Data set 7	Functional annotations of *C. friedae*	Lis file (.lis)	Figshare, 10.6084/m9.figshare.25108073. [26]
Data set 8	Repeat annotations of *O. transversa*	Gff file (.gff)	Figshare, 10.6084/m9.figshare.25108073. [26]
Data set 9	Repeat annotations of *C. friedae*	Gff file (.gff)	Figshare, 10.6084/m9.figshare.25108073. [26]
Data set 10	Annotation of mtDNA of *O. transversa*	Text file (.txt)	Figshare, 10.6084/m9.figshare.25108073. [26]
Data set 11	Annotation of mtDNA of *C. friedae*	Text file (.txt)	Figshare, 10.6084/m9.figshare.25108073. [26]

In addition, we collected RNA-seq data to assist gene annotation with the remaining samples after genome sequencing. Total RNA was extracted from the whole bodies of two *O. transversa* gynes, 15 *C. friedae* small workers and 10 *C. friedae* middle workers by the Trizol method. Then, the three RNA samples were subjected to RNA-seq library construction and paired-end sequencing with the DNBSEQ-T1 system at China National GeneBank (Shenzhen, China). Finally, we obtained 42.2 Gb and 43.2 Gb of RNA-seq data for the small workers and middle workers of *C. friedae*, respectively, and 38.0 Gb for the gynes of *O. transversa* (Table 1, Data file 2).

The PacBio HiFi reads were assembled by Wtdbg2 (v2.5) [13] with the mode optimal for HiFi data (parameters: -x ccs), which yielded a draft genome assembly of 339 Mb for *O. transversa* and 233 Mb for *C. friedae*, respectively (Table 1, Data set 3). The *O. transversa* assembly comprised 6,442 contigs with an N50 length of 101.7 kb and a GC content of 41%, while the *C. friedae* assembly contained 3,302 contigs with an N50 length of 159.7 kb and a GC content of 35% (Data file 3). Minimap2 (v2.1) [14] was used to align the HiFi reads to the assembled genomes, which reported an alignment rate of 99.5% for *O. transversa* and 94.3% for *C. friedae*. BUSCO (v5.3.2) [15] assessment based on the hymenopteran gene database revealed that both genome assemblies had a completeness score over 90% (Data file 4). The consensus quality value (QV) assessed by Merqury (v1.3) [16] with the HiFi data was 38.3 for *O. transversa* and 46.8 for *C. friedae*, respectively. In addition, more than 99% of the genomic positions in the two assemblies were covered by at least three HiFi reads and ~ 98% covered by at least five HiFi reads (Data file 5). Taken together, these metrics support a good completeness and high accuracy of the *O. transversa* and *C. friedae* genome assemblies.

Protein-coding genes were predicted using GeMoMa (v1.9), which utilizes homologous and RNA-seq evidence to accurately predict gene models [17]. Specifically, RNA-seq reads were first aligned to the genome using Hisat2 (v2.2.1) [18], followed by reference-based transcriptome assembly using StringTie2 (v2.1.4) [19] and open reading frame prediction using TransDecoder (v5.7.1) [20]. Then the transcriptome-derived gene models of *O. transversa* and *C. friedae* were combined with the gene models of *C. floridanus*, *Atta cephalotes*, *Ooceraea biroi*, *Nasonia vitripennis* and *Tribolium castaneum* to serve as homologous evidence for GeMoMa. In the meanwhile, RNA-seq derived splice junctions from Hisat2 alignments were applied by GeMoMa to refine the exon-intron boundaries (Table 1, Data set 4,5). In total, 10,892 and 11,296 protein-coding genes were identified in the genomes of *O. transversa* and *C. friedae*, respectively. BUSCO assessment with the hymenopteran gene database reported a completeness score around 90% for both gene sets (Data file 6). In addition, homologous searches against databases of InterPro, UniProtKB, NCBI nr, and KEGG could assign putative functional annotations for more than 95% of the protein-coding genes (Data set 6,7).

The annotation of repetitive elements (Table 1, Data set 8,9) was conducted by RepeatMasker (v4.1.5) [21], RepeatModeler (v2.0.4) [22], and Tandem Repeats Finder (v4.07b) [23]. The total non-redundant length of the *O. transversa* repeats resulted from these methods was 149.4 Mb, accounting for 44.0% of the assembled genome size, while the total length of *C. friedae* repeats was 49.7 Mb which represented 21.3% of the genome size (Data file 7).

The mitochondrial genomes of the two ants were assembled by MitoZ (v2.2) [24]. Mitochondrial gene annotation was carried out using the *annotate* function of MitoZ (--clade Arthropoda) and the online server of MITOS2 (--code Invertebrate (5), --refseqver RefSeq63 Metazoa) [25], followed by manual check of each gene locus. The total lengths of the *O. transversa* and *C. friedae* mitochondrial genome assemblies were 16.1 kb and 18.8 kb, respectively, with all the expected mitochondrial genes (13 protein-coding genes, 22 tRNA genes, and 2 rRNA genes) identified (Table 1, Data set 10,11).

## Limitations

The genome assemblies of the two ant species are still fragmented, which disable the study of chromosome-level rearrangements or structural variation. The incorporation of Hi-C sequencing data to achieve chromosome-level assemblies is expected to overcome this limitation in the future. In addition, the collection of transcriptome data from more castes and developmental stages are required to further improve the gene annotations. Nevertheless, regardless of these limitations, we anticipate that the draft genome assemblies together with the genome annotations generated in this study are valuable for phylogenomics and comparative genomics of ants and hymenopteran insects.

## Data Availability

The PacBio HiFi data and RNA-seq data generated in this study are deposited in the CNGB Nucleotide Sequence Archive under accession no. CNP0005508 and NCBI under Project accession no. PRJNA1114375. And other data is provided within the manuscript files.

## References

[1] Hölldobler B, Wilson EO. The Superorganism: The Beauty, Elegance, and Strangeness of Insect Societies. W. W. Norton & Company. 2009;299:106.

[2] Romiguier J, Borowiec ML, Weyna A, Helleu Q, Loire E, La Mendola C, Rabeling C, Fisher BL. Ward PS and Keller L. Ant phylogenomics reveals a natural selection hotspot preceding the origin of complex eusociality. Curr Biol. 2022;32:2942-7.e4. 10.1016/j.cub.2022.05.001 35623348

[3] AntCat. Accessed at. An Online Catalog of the Ants of the World. http://antcat.org/. 2024.

[4] Boomsma JJ, Brady SG, Dunn RR, Gadau J, Zhang G (2017). The Global Ant Genomics Alliance (GAGA). Myrmecol News.

[5] Tsutsui ND, Suarez AV, Spagna JC, Johnston JS (2008). The evolution of genome size in ants. BMC Evol Biol.

[6] Guénard BS, Dunn RR (2012). A checklist of the ants of China. Zootaxa.

[7] Yamane S (2009). Odontoponera denticulata (F. Smith) (Formicidae: Ponerinae), a distinct species inhabiting disturbed areas. ARI.

[8] Martinho G. Accessed at Odontoponera - Care Guide and Ecology. https://www.thewildmartin.com/ant-ecology/odontoponera-care-guide-and-ecology. 2021.

[9] Wen X-L, Wen P, Dahlsjö CAL (2017). Sillam-Dussès D and Šobotník J. breaking the cipher: ant eavesdropping on the variational trail pheromone of its termite prey. Proc Biol Sci.

[10] Ke Y-L, Tian W-J, Zhuang T-Y, Wang C-X, Liang M-F (2013). Nest Architecture of four Ponerine ant species (Formicidae, Ponerinaeand Organisms Present in their nests. J South China Agricultural Univ.

[11] Rossi N, Feldhaar H. Carpenter Ants. In: Starr CK, editor. Encyclopedia of Social insects. Springer Cham; 2020. pp. 157–61.

[12] Terayama M (1999). The ant genus Camponotus Mayr (Hymenoptera: Formicidae) in Japan. Mem Myrmecological Soc Japan.

[13] Ruan J, Li H (2019). Fast and accurate long-read assembly with wtdbg2. Nat Methods.

[14] Li H, Birol I (2018). Minimap2: pairwise alignment for nucleotide sequences. Bioinformatics.

[15] Seppey M, Manni M, Zdobnov EMBUSCO. Assessing Genome Assembly and Annotation Completeness. In, editors. Methods in molecular biology. 2019. pp. 227 – 45. 10.1007/978-1-4939-9173-0_14 31020564

[16] Arang Rhie BPW (2020). Sergey Koren and Adam M. Phillippy. Merqury: reference-free quality, completeness, and phasing assessment for genome assemblies. Genome Biol.

[17] Keilwagen J, Hartung F, Grau J, GeMoMa. Homology-based gene prediction utilizing intron position conservation and RNA-seq data. Methods Mol Biol. 2019;1962:161–77. 10.1007/978-1-4939-9173-0_9 31020559

[18] Kim D, Paggi JM, Park C, Bennett C, Salzberg SL (2019). Graph-based genome alignment and genotyping with HISAT2 and HISAT-genotype. Nat Biotechnol.

[19] Kovaka S, Zimin AV, Pertea GM, Razaghi R, Salzberg SL, Pertea M (2019). Transcriptome assembly from long-read RNA-seq alignments with StringTie2. Genome Biol.

[20] Haas BJ. Accessed at. Find Coding Regions Within Transcripts. https://github.com/TransDecoder/TransDecoder. 2023.

[21] Robert H, Arian S. A program screens DNA sequences for interspersed repeats and low complexity DNA sequences. https://www.repeatmasker.org. Accessed at 2023.

[22] Robert H, Arian S. A de novo transposable element (TE) family identification and modeling package. https://www.repeatmasker.org/RepeatModeler. Accessed at 2023.

[23] Benson G (1999). Tandem repeats finder: a program to analyze DNA sequences. Nucleic Acids Res.

[24] Meng G, Li Y, Yang C, Liu S (2019). MitoZ: a toolkit for animal mitochondrial genome assembly, annotation and visualization. Nucleic Acids Res.

[25] Donath A, Jühling F, Al-Arab M, Bernhart SH, Reinhardt F, Stadler PF, Middendorf M, Bernt M (2019). Improved annotation of protein-coding genes boundaries in metazoan mitochondrial genomes. Nucleic Acids Res.

[26] Liu J-L, Xiong Z-J, Pan Y-L, Zhao J. Draft genome assemblies of the ponerine ant Odontoponera transversa and the carpenter ant Camponotus friedae (Hymenoptera: Formicidae). figshare. 2024. 10.6084/m9.figshare.25108073. 10.1186/s12863-024-01253-7 PMC11251112 39009995

[27] Liu J-L, Xiong Z-J, Pan Y-L, Zhao JNCBI, Sequence Read. 2024. NCBI https://identifiers.org/ncbi/insdc.sra:SRP509859.

[28] Liu J-L, Xiong Z-J, Pan Y-L, Zhao JCNGB, Sequence Read. 2024. CNGB https://db.cngb.org/search/project/CNP0005508.

[29] Liu J-L, Xiong Z-J, Pan Y-L, Zhao J. Genome assemblies of Odontoponera transversa and Camponotus friedae. 2024. NCBI https://identifiers.org/ncbi/bioproject:PRJNA1114375. 10.1186/s12863-024-01253-7 PMC11251112 39009995

[30] Liu J-L, Xiong Z-J, Pan Y-L, Zhao J. Genome Assemblies. 2024. CNGB https://db.cngb.org/search/project/CNP0005508.

